# Mediators and Patterns of Muscle Loss in Chronic Systemic Inflammation

**DOI:** 10.3389/fphys.2018.00409

**Published:** 2018-04-24

**Authors:** Sandra Pérez-Baos, Iván Prieto-Potin, Jorge A. Román-Blas, Olga Sánchez-Pernaute, Raquel Largo, Gabriel Herrero-Beaumont

**Affiliations:** Bone and Joint Research Unit, Service of Rheumatology, IIS-Fundación Jiménez Díaz, Autonomous University of Madrid, Madrid, Spain

**Keywords:** skeletal muscle, turnover, anabolism, catabolism, sarcopenia, myokines, inflammation

## Abstract

Besides its primary function in locomotion, skeletal muscle (SKM), which represents up to half of human's weight, also plays a fundamental homeostatic role. Through the secretion of soluble peptides, or myokines, SKM interacts with major organs involved in metabolic processes. In turn, metabolic cues from these organs are received by muscle cells, which adapt their response accordingly. This is done through an intricate intracellular signaling network characterized by the cross-talking between anabolic and catabolic pathways. A fine regulation of the network is required to protect the organism from an excessive energy expenditure. Systemic inflammation evokes a catabolic reaction in SKM known as sarcopenia. In turn this response comprises several mechanisms, which vary depending on the nature of the insult and its magnitude. In this regard, aging, chronic inflammatory systemic diseases, osteoarthritis and idiopathic inflammatory myopathies can lead to muscle loss. Interestingly, sarcopenia may persist despite remission of chronic inflammation, an issue which warrants further research. The Janus kinase/signal transducer and activator of transcription (JAK/STAT) system stands as a major participant in muscle loss during systemic inflammation, while it is also a well-recognized orchestrator of muscle cell turnover. Herein we summarize current knowledge about models of sarcopenia, their triggers and major mediators and their effect on both protein and cell growth yields. Also, the dual action of the JAK/STAT pathway in muscle mass changes is discussed. We highlight the need to unravel the precise contribution of this system to sarcopenia in order to design targeted therapeutic strategies.

Skeletal muscle (SKM) is a vast organ, which accounts for 40% of total weight in non-obese population (Janssen et al., 2000). The high metabolic activity of muscle cells, or myocytes, not only provides the necessary contraction for locomotion, but also fuels other organs' functions. Through the secretion of soluble peptides called myokines, the SKM interacts with surrounding fat, bones and skin, as well as with principal organs, including the cardiovascular system, brain, digestive tract and glands (Hartwig et al., 2014; Giudice and Taylor, 2017). In addition, some myokines exhibit autocrine/paracrine actions in the muscle, thereby helping sustain its normal growth (Pedersen and Febbraio, 2008). Considering the active interplay between SKM and other tissues, the impact of physical inactivity on general metabolism can be envisaged. In short, lack of exercise results in a lower insulin sensitivity, changes in the postprandial lipid profile, and accumulation of visceral adipose tissue (Pedersen and Febbraio, 2012). These effects are conceived to be the result of an evolutionary positive selection of pro-inflammatory pathways as well as of genes favoring gluconeogenesis, insulin resistance and fat storage. While formerly considered advantageous traits, in populations exposed to famines and epidemics, they have turned into a burden in modern ages, furthering cardiovascular diseases, because of lifestyle changes (Tuomilehto et al., 2001; Nocon et al., 2008).

Not only does SKM adjust the individual's metabolic activities, but it also suffers the consequences of perturbations in the systemic milieu. The SKM response to environmental cues is regulated by the hypothalamus, which integrates endocrine and immune signals, as well as information about physical activity and nutritional state (Clegg et al., 2013). Thus, levels of some nutrients –like glucose, fatty acids and amino acids– along with leptin and additional adiposity-related hormones (Roh et al., 2016) act as inputs for the elaboration of brain responses controlling energy expenditure, food intake, insulin secretion and glucose/fatty acid turnover in SKM (Roh et al., 2016). Muscle homeostasis requires fine-tuning of both protein turnover and cell growth in order to adapt its response to particular needs of the individual, without affecting muscle mass balance.

There is a dramatical shift in SKM homeostasis toward muscle loss during chronic inflammation. It is thought that an overacting immune system can divert energy expenditure and lead to a shortage of stored reserves affecting general metabolism (Straub, 2017). In spite of their relevance, these collateral effects are often forgotten in the assessment of prevalent conditions, while there is an increasing need of accurately measuring SKM response to stress induced by a variety of insults. However, the pathogenesis of systemic inflammation-related SKM damage, which has been termed cachexia or more accurately sarcopenia, has not until recently been understood. In this regard, sarcopenia can be observed in chronic debilitating diseases, as well as in typical inflammatory conditions, like rheumatoid arthritis, or in the proximity of injured joints, as in the case of osteoarthritis. It can also be the result of primary SKM autoimmune diseases. In the same way that the precipitating conditions are quite distinct in nature, so is the pattern of sarcopenia associated to them, while it also depends on the severity of the injury. All these factors make of sarcopenia a complex entity influencing general health which should be addressed in the therapeutic management of diseases.

This article gives an overview on triggers and mediators of SKM protein synthesis and cell turnover, especially focusing on clinical situations associated to muscle loss. The impact of systemic inflammation on muscle mass is discussed, looking into the molecular signals which disrupt muscle homeostasis in the different models of sarcopenia. In particular, the dual role of the Janus kinase/signal transducer and activator of transcription (JAK/STAT) pathway is underlined.

## Muscle protein turnover

**Figure 1:**
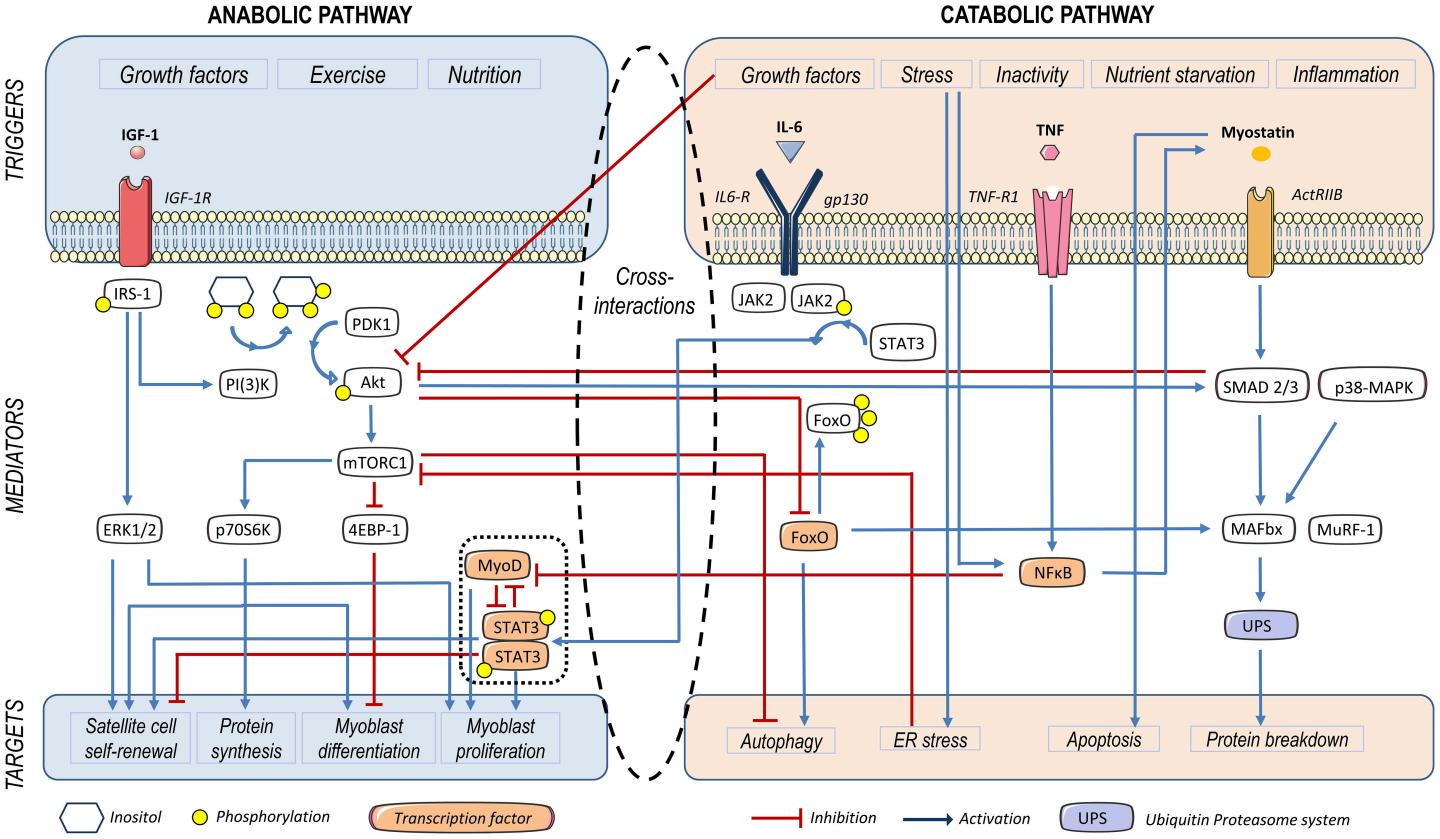
Triggers and mediators participating in protein and cell turnover of the skeletal muscle. Insulin growth factor (IGF)-1, leads to insulin receptor substrate-1 (IRS-1) phosphorylation. Subsequently, Akt becomes phosphorylated and activates the mammalian target of rapamycin (mTOR)-C1, thus contributing to protein synthesis and myoblast proliferation. Furthermore, Akt represses Forkhead O (FoxO), a factor that activates autophagy and the ubiquitin proteasome system (UPS) involved in protein catabolism. IRS-1 also activates the extracellular signal–regulated kinase (ERK), leading to satellite cell self-renewal and myoblast proliferation. IL-6-dependent activation of STAT-3 contributes to myogenic differentiation and SC proliferation in homeostatic conditions. Myostatin (MSTN) activates Smad2/3, which activates the ubiquitin proteasome system (UPS). Smad2/3 also inhibits the PI3K/Akt/mTOR signaling pathway, thus diminishing myoblast differentiation and protein synthesis. UPS is also activated by Forkhead O (FoxO), p38 mitogen activated protein kinases (MAPK) and nuclear factor κB (NFκB), which also induces a decay in MyoD mRNA, thus leading to a decrease in myoblast differentiation. IL-6-induced JAK2/STAT-3 signaling impairs myogenesis in a catabolic scenario.

The major driver of SKM anabolic activity is the phosphatidylinositol 3-kinases (PIK3)/Akt signaling pathway. Along with exercise, insulin and insulin growth factor (IGF)-1 can induce phosphorylation of the insulin receptor substrate (IRS)-1 through the binding of specific receptors, subsequently activating PI3K (Sandri et al., 2013). In turn, the end product phosphatidylinositol-3, 4, 5 -trisphosphate (PI3P) facilitates membrane anchorage of Akt and its phosphorylation by 3′-phosphoinositide-dependent protein kinase-1 (PDK-1), upon which Akt enables activation of the mechanistic target of rapamycin (mTOR). The latter acts as downstream effector of the anabolic pathway through both the stimulation of ribosomal protein S6 kinase beta-1 (RS6K, or 70S6K), and the inhibition of 4E-binding protein 1 (4E-BP1) (Glass, 2003) (Figure 1). As we will discuss, Akt also shuts down the expression of forkhead O (FoxO), a transcriptional activator of muscle-specific E3 ubiquitin ligases involved in protein catabolism (Mammucari et al., 2007; Schiaffino et al., 2013). On the other hand, testosterone can induce muscle hypertrophy through the PI3K/Akt/mTOR pathway or also using androgen receptors (Basualto-Alarcón et al., 2013; Hughes et al., 2016).

Conversely, myostatin (MSTN) –or growth differentiation factor-8 (GDF-8)– is a member of the transforming growth factor β (TGF-β) family, and a negative regulator of muscle growth. The MSTN-Smad2/3 route inhibits Akt-dependent protein synthesis and growth of mature muscle cells (Morissette et al., 2009; Schiaffino et al., 2013; Brooks and Myburgh, 2014; Retamales et al., 2015). This myokine binds to activin type 2 receptors (ACVR2) and promotes cachexia-related catabolism, via FoxO-dependent induction of atrogenes (McFarlane et al., 2006) (Figure 1). As it has been shown both in rodents and humans, training acts as a MSTN repressor. This fact has been put in relationship with the benefit of endurance exercise on metabolism. On the other hand, both muscle and serum of obese individuals appear to be comparatively enriched in MSTN (Hittel et al., 2009).

### Regulatory networks of protein turnover

The concurrence of pro/anti-anabolic and pro/anti-catabolic factors, not only within the muscle milieu but also in the systemic environment, determines the yield of muscle turnover (Reed et al., 2012; Schiaffino et al., 2013). Usually, up-regulation of protein synthesis is accompanied by a reduction of protein degradation (Schiaffino et al., 2013). Likewise, under catabolic stress, a well-orchestrated network blocks new protein synthesis in order to limit energy expenditure. Nonetheless, if conditions are favorable, the amino acids released during proteolysis will boost new protein synthesis (Tran et al., 2007; Zoncu et al., 2011; Perl, 2015).

The role of microRNAs in muscle anabolic fine-tuning is noteworthy. MicroRNAs are small non-coding RNAs which act as negative regulators of gene expression. Muscle specific microRNAs, known as myomiRs, target myostatin, TGF-β and Akt-dependent pathways. In turn, these factors modify the expression rate of different myomiRs (Butz et al., 2012; Hitachi and Tsuchida, 2014; Jung and Suh, 2015). Their isolation from muscle exosomes suggests that myomiRs can be released and exert autocrine/paracrine actions in surrounding cells, in this way facilitating a synchronous muscle growth response (Demonbreun and McNally, 2017; Fry et al., 2017).

## Cell growth

### Myogenesis

Although anabolic factors have commonly a role in myogenesis, processes of cell growth and protein synthesis have to be considered as independent parts of the SKM metabolism. In particular, myogenesis accounts for the capacity of cell renewal and differentiation during repair processes.

This capacity is based on the existence of a niche of quiescent myogenic cells, known as satellite cells (SC), between the outer coat of sarcolemma and the basal lamina of myofibers (Morgan and Partridge, 2003). These cells are characterized by the expression of paired box transcription factor (Pax) 7 and myogenic factor (Myf) 5 and have a crucial role in self-renewal of SKM. Upon injury, SC undergo proliferation and differentiation into myoblasts (Pax7+/Myf5+/MyoD+), which eventually lose expression of Pax7 and upregulate myogenin (Pax7-/Myf5+/MyoD+/Myogenin+). The latter determines cessation of cell proliferation and progression to terminal differentiation, maturation and fusion of cells into new myofibers (Morgan and Partridge, 2003).

The JAK/STAT pathway and its triggering cytokine IL-6 stand as key myogenic factors. The intracellular network exerts a relevant homeostatic role in healthy SKM. Normal proliferating myoblasts have been found to exhibit high levels of phospho-STAT-3, both in vivo (Kami and Senba, 2002) and in culture (Spangenburg and Booth, 2002; Yang et al., 2009). According to the studies performed by Hoene and co-workers in primary mouse myoblasts and in C2C12 cells, the activation of STAT-3-SOCS-3 in response to an autocrine action of IL-6 is needed for their differentiation (Hoene et al., 2013). In the same way, IL-6 stimulation of STAT-3 is required for SC proliferation following SKM overload. Moreover, not only does IL-6 stimulate myogenesis, but it also exerts some key metabolic actions (Pedersen and Febbraio, 2008; Muñoz-Cánoves et al., 2013) and blocks the production of pro-apoptotic cytokines like tumor necrosis factor alpha (TNF) (Schindler et al., 1990). Altogether, these actions place IL-6 as a pivotal myokine released from muscle cells upon exercise.

Myoblast proliferation is subject to a tight control, which prevents alterations in muscle mass volume during repair processes (Verdijk et al., 2007; McFarlane et al., 2011; Fry et al., 2015; Demonbreun and McNally, 2017). Experiments conducted in the rodent C2C12 and L6 myoblast cell lines have shown that IGF-1 plays opposite roles at distinct stages of myocyte differentiation. While the peptide initially exerts a mitogenic effect, it inhibits growth and promotes cell differentiation at later phases (Engert et al., 1996; Florini et al., 1996). In turn, these polar activities are driven by different signaling pathways. Extracellular signal-regulated kinase (ERK) appears to be involved in cell proliferation (Rosenthal and Cheng, 1995; Coolican et al., 1997), whereas PI3K/Akt/p70S6K has been shown to mediate IGF-1-induced myogenin expression (Xu and Wu, 2000) (Figure 1). Similarly, the JAK/STAT pathway may promote either proliferation or differentiation depending on the molecular isoforms involved in its activation, as we will discuss (Sun et al., 2007; Wang et al., 2008; Diao et al., 2009).

## Regulatory catabolic processes

Metabolic disturbances and stress trigger numerous repair mechanisms in SKM aimed at restoring homeostasis. Intracellular proteolytic complexes, including calpains, the endoplasmic reticulum (ER) stress response, caspase cascades, the autophagic machinery and the ubiquitin-proteasome system (UPS) can participate in such response. Dysregulation and/or perpetuation of these reparative mechanisms may result in muscle wasting, either by increasing proteolysis or initiating muscle cell apoptosis.

### The ubiquitin-proteasome system (UPS)

The UPS accounts for the principal mechanism of protein degradation in muscle cells (Glass, 2010; Milan et al., 2015). Two muscle-specific E3 ligases, muscle ring finger-1 (MuRF-1) and muscle atrophy F-box protein (MAFbx) –or atrogin-1– (Schiaffino et al., 2013) have been identified and their expression is commonly used as a marker of UPS activity in secondary sarcopenia models (Milan et al., 2015). UPS is regulated by FoxO (Sandri et al., 2004; Tournadre et al., 2017), p38 mitogen activated protein kinase (MAPK) (Zhang and Li, 2012), STAT-3 (Bonetto et al., 2012) and nuclear factor κB (NFκB) (Cai et al., 2004). Furthermore, STAT-3 and NFκB down-regulate MyoD expression, and, consequently, decrease myoblast differentiation (Guttridge et al., 2000) (Figure 1). While FoxO is blocked by Akt, the other mediators are activated in response to pro-inflammatory molecules (Schiaffino et al., 2013). Notably, insulin peripheral resistance, which shuts the IGF1-Akt pathway down, also raises UPS activity (de Alvaro et al., 2004; Brown et al., 2009) (Figure 1).

### Autophagy

Autophagy is carried out by a cascade of proteolytic reactions which degrade and recycle malfunctioning organelles, proteins and other cytoplasmic molecules, using the lysosomal machinery and a multimolecular complex (Mizushima and Komatsu, 2011). This highly conserved mechanism of homeostasis allows cells to survive under stress conditions (Cuervo, 2008). In addition, defects in autophagy associate with muscle disease and inflammation (Levine and Kroemer, 2008). However, over-induction of autophagy is also responsible for muscle loss. Up to 35 different autophagy-related genes (ATG) encoding the autophagy machinery have been found. The inhibition of mTOR complex (mTORC)1 activates the initiation steps of autophagy, inducing autophagosome nucleation and substrate recognition. This is followed by the recruitment of different ATG products that facilitate protease digestion of the cargo (Portal-Núñez et al., 2015). Likewise, muscle mass can also be regulated by mitochondrial dysfunction, ER stress or myocyte apoptosis (Nagaraju et al., 2005; Busquets et al., 2007; Nogalska et al., 2007; Marzetti et al., 2009, 2010; Deldicque, 2013).

### Endoplasmic reticulum (ER) stress

ER and mitochondria cooperate in SKM homeostasis. Disturbances of their balance can be induced by protein misfolding, starvation or energy deprivation, and lead to an ER stress response. Consequently, ER activates the so-called ‘unfolded protein response’, consisting in the upregulation of chaperones and other enzymes that participate in adequate protein folding. If the insult persists, the stress response can be unable to maintain cell homeostasis and give rise to the activation of NF-κB and other inflammatory pathways. Uncontrolled ER stress eventually lead to myocyte death by mechanisms which include apoptosis, autophagy and necrosis (Deldicque, 2013). Furthermore, it has been proposed that ER stress could indirectly contribute to muscle wasting through the blockage of mTORC1, thus creating a state of anabolic resistance (Deldicque, 2013).

### Apoptosis

Myofiber loss accounts for the key process leading to sarcopenia during aging, skeletal muscle immobilization, muscular dystrophy and other inflammatory conditions (Dupont-Versteegden, 2006). The specific mechanisms of cell death in SKM remain largely unknown and will not be addressed in this review. Noteworthy, Dupont-Versteegden and co-workers suggested that classical apoptotic pathways could be of little relevance in muscle. Alternatively, additional molecules might exert a key role in triggering regulatory cell death processes (Dupont-Versteegden, 2006). Of note, being myofibers multinucleated syncytia, nuclear elimination does not necessarily carry cell destruction. In this regard, muscle loss can rely on loss of myonuclei or of their associated cytoplasmic domains (Allen et al., 1999).

## Sarcopenia, the SKM response to systemic inflammatory stress

Systemic inflammation affects all body systems and organs, including the SKM. Muscle response can follow different patterns, namely primary sarcopenia, secondary sarcopenia, and those respectively found in osteoarthritis and in idiopathic inflammatory myopathies. These four inflammation-related clinical settings of muscle wasting are summarized in Table 1 and will be addressed below.

**Table 1:** Inflammatory description and altered molecular mediators in four clinical settings related to muscle wasting. −/+: absent / low; +: mild; +++: high.

	**Primary sarcopenia**	**Secondary sarcopenia**	**OA sarcopenia**	**Idiopathic inflammatory myopathies**	**Authors**
Intensity of chronic inflammation	Low grade [1,2]	High grade [3,4,5,6]	Low grade [7,8,9]	High grade [10]	Beyer et al., 2012 ^[1]^; Clegg et al., 2013 ^[2]^; Dalakas, 2010 ^[10]^; Krabbe et al., 2004 ^[7]^; Morley et al., 2006 ^[3]^; Robinson et al., 2016 ^[8]^; Roubenoff, 2014 ^[4]^; Summers et al., 2010 ^[5]^; von Haehling et al., 2016 ^[6]^; Scanzello and Loeser, 2015 ^[9]^
Systemic inflammatory markers	−/+ [1,2,7,23,24,25]	+++ [3,4,5,6] Adipokines [26,27]	+ [8,14,15,16] Adipokines [26,27]	−/+ [28,29]	Beyer et al., 2012 ^[1]^; Clegg et al., 2013 ^[2]^; Creus et al., 2009 ^[23]^; Gómez et al., 2015 ^[14]^; Hanisch and Zierz, 2015 ^[28]^; Herrero-Beaumont et al., 2009 ^[15]^; Hotamisligil, 2006 ^[16]^; Krabbe et al., 2004 ^[7]^; Maggio et al., 2006 ^[24]^; Malik et al., 2016 ^[29]^; Morley et al., 2006 ^[3]^; Roubenoff, 2014 ^[4]^; Scotece et al., 2011 ^[26]^; Scotece et al., 2017 ^[27]^; Summers et al., 2010 ^[5]^; Varadhan et al., 2014 ^[25]^; von Haehling et al., 2016 ^[6]^
Local inflammatory markers	IL-1 IL-6, TNF [7,23,24,30]	IL-1 [20], IL-6, TNF [20,31], IFN-γ [20,31]	IL-6?	IL-1, TNF [32,33] IFN-γ [33]	Creus et al., 2009 ^[23]^; Krabbe et al., 2004 ^[7]^; Little et al., 2017 ^[20]^; Loell and Lundberg, 2011 ^[32]^; Maggio et al., 2006 ^[24]^; de Oliveira Nunes Teixeira et al., 2013 ^[30]^; Tews and Goebel, 1996 ^[33]^; Varadhan et al., 2014 ^[30]^
Major type of metabolic process	Anti-anabolism [11,12]	Pro-catabolism [13]	Anti-anabolism [7,8,9] Pro-catabolism [8,14,15,16]	?	Ali and Garcia, 2014^[11]^; Drummond et al., 2009 ^[12]^; Gómez et al., 2015 ^[14]^; Herrero-Beaumont et al., 2009 ^[15]^; Hotamisligil, 2006 ^[16]^; Krabbe et al., 2004 ^[7]^; Masuko, 2014 ^[13]^; Robinson et al., 2016 ^[8]^; Scanzello and Loeser, 2015 ^[9]^
Major anabolic / catabolic signaling mediators	↑MSTN [17] ↓IGF/Akt/mTOR [18]	↑MSTN [19] ↓MSTN [20] ↑UPS [20,21]	?	↑MSTN [22] ↓IGF/Akt/mTOR? ↑UPS?	Castillero et al., 2009a ^[21]^; Little et al., 2017 ^[20]^; McFarlane et al., 2006 ^[19]^; Nogalska et al., 2007 ^[22]^; Sattler, 2013 ^[18]^; Vermeulen et al., 2017 ^[17]^
Transcription factors representing a pivotal link with sarcopenia	NFκB [7,23,24,30] STAT [36,37] FoxO [34] MyoD [35]	NFκB [38,39,40] STAT [41,42] FoxO [19] MyoD [21,38,39,40] Myogenin [21,38,39,40]	NFκB [43] STAT [44,45]	NFκB [46] STAT [47]	Bonetto et al., 2011 ^[41]^; Bonetto et al., 2012 ^[42]^; Brack et al., 2005 ^[35]^; Cai et al., 2004 ^[38]^; Castillero et al., 2009a ^[21]^; Castillero et al., 2009b ^[39]^; Creus et al., 2009 ^[23]^; Guttridge et al., 2000 ^[40]^; Illa et al., 1997 ^[47]^; Krabbe et al., 2004 ^[7]^; Levinger et al., 2011a ^[43]^; Maggio et al., 2006 ^[24]^; Malemud, 2017 ^[45]^; McFarlane et al., 2006 ^[19]^; Nogalska et al., 2007 ^[46]^; Price et al., 2015 ^[36]^; Sandri et al., 2013 ^[30]^; Santos et al., 2011 ^[44]^; Tierney et al., 2014 ^[37]^; Varadhan et al., 2014 ^[30]^
Changes in fat mass accompanying sarcopenia	Increase [48]	Decrease (sometimes stable or increase) [49,50,51,52]	Increase (sarcopenic obesity) [52]	Increase [53]	Cleary et al., 2015 ^[53]^; Giles et al., 2008 ^[49]^; Lee et al., 2012 ^[52]^; Lemmey et al., 2009 ^[50]^; Tournadre et al., 2017 ^[50]^; Volpi et al., 2004^[48]^; Walsmith and Roubenoff, 2002 ^[51]^
Association with metabolic syndrome	No	Yes [3,4,5,6]	Yes [7,8,9,8,14,15,16,52]	No	Gómez et al., 2015 ^[14]^; Herrero-Beaumont et al., 2009 ^[15]^; Hotamisligil, 2006 ^[16]^; Krabbe et al., 2004 ^[7]^; Lee et al., 2012 ^[52]^; Morley et al., 2006 ^[3]^; Robinson et al., 2016 ^[8]^; Roubenoff, 2014 ^[4]^; Scanzello and Loeser, 2015 ^[9]^; Summers et al., 2010 ^[5]^; von Haehling et al., 2016 ^[6]^

### Primary sarcopenia

The condition known as primary sarcopenia (PS) is associated with the aging and the frailty syndrome and is defined as a progressive and generalized loss of SKM mass and/or strength leading to a significant functional impairment (Cruz-Jentoft et al., 2010). This muscle response is considered to be the consequence of aging/disease interactions at multiple systems (Cruz-Jentoft et al., 2010). Thus, PS identifies a poor health status frequently associated with disability, increasing risk of falls and fractures, and potentially dragging the elderly to dependence. On these grounds, its appearance leads to a decreased life expectancy (Cruz-Jentoft et al., 2010).

This type of sarcopenia represents a failure in muscle anabolic processes. In addition, it is likely that a mild persistent inflammatory status could play a role in its pathogenesis, promoting a catabolic scenario(Ali and Garcia, 2014).

Morphologically, atrophy of type II (fast-twitch, highly ATP-consuming) fibers can be observed, following a loss of myocyte proteins, organelles and cytoplasm size (Muscaritoli et al., 2010), and the accumulation of muscle fat. The origin of PS has been put in relationship with age-dependent functional changes in mitochondria, ER, cells and tissues. Furthermore, as suggested by studies conducted in aged mice, the niche of SC could decline with age and be insufficient for nuclear replacement (Brack et al., 2005). Additional factors are related to lack of mobility, neurodegeneration, nutritional deficiencies and hormone decrease. Progressive testosterone deficiency increases peripheral resistance to insulin and IGF-1 –both potent activators of the Akt/mTOR pathway– yielding a lower synthesis and a higher degradation of muscle proteins, through the activation of FoxO (Sandri et al., 2004). Likewise, an age-dependent impairment in the GH/IGF-1 axis has been reported. A fall in gene expression of GH receptors in the elderly inversely correlates with serum MSTN levels (Perrini et al., 2010). This fact could hamper both synthetic processes and cell renewal (Taylor et al., 2001) –and the same effects could be expected from the lowering of sex hormone levels –. Of all myokines, IGF-1 is regarded as a pivotal mediator of muscle growth because of its effect on SC proliferation, and its concentration is inversely associated with the development of PS. However, recent experiments have suggested that deficiency of GH/IGF-1 could increase longevity in animals (Sattler, 2013). Indeed, some human studies have also drawn controversial results, a matter which has diverted the attention from the GH/IGF-1 axis to MSTN up-regulation, as the cornerstone of the anti-anabolic response of PS.

On the other hand, although clearly regarded as an anabolic resistant process, there is also an age-related low-grade chronic inflammation that may contribute to muscle wasting in PS (Clegg et al., 2013) through the up-regulation of pro-inflammatory cytokines, like TNF, IL-1 and IL-6 (Krabbe et al., 2004; Maggio et al., 2006; Drummond et al., 2009; Ali and Garcia, 2014). This view is supported by the finding of differential levels of serum biomarkers between active and non-active elders, with an enhancement of IL-8, myeloperoxidase and TNF in the latter group (Marzetti et al., 2014).

Increased levels of IL-6 appear to be particularly associated to a higher disability in the elderly (Barbieri et al., 2003; Maggio et al., 2006). In spite of its homeostatic role in SKM, both driving myogenesis and mediating IGF-I anabolic actions (Barbieri et al., 2003; Maggio et al., 2006; Mammucari et al., 2007; Schiaffino et al., 2013), over-expression of IL-6 is known to impair myocyte functions (Roubenoff, 2014). Indeed, its levels along with those of TNF-R1 out of a group of 15 different NF-kB-dependent molecules, have been found to be the best predictor of mortality in the elderly (Varadhan et al., 2014). Altogether this suggests that the inflammatory response could account for a therapeutic target in PS.

### Secondary sarcopenia (SS)

Secondary sarcopenia (SS) occurs in the context of chronic illnesses, like cancer, renal/respiratory failure or inflammatory diseases. A paradigm of this SKM response is rheumatoid sarcopenia –or sarcopenia during rheumatoid arthritis– (RS) (Morley et al., 2006; von Haehling et al., 2016). Sarcopenia is a prominent feature of the more generalized rheumatoid cachexia syndrome, thus termed because it targets major organs and immune cells along with the SKM, leading to a profound loss of cell mass in all these tissues. In this regard, two types of cachexia have been associated to RA; the ‘classic’ type –which resembles those found in cancer and AIDS– and shows both muscle and fat mass loss, and the more typical ‘rheumatoid’ type –which results in a reduced muscle mass but an increase in fat volume– (Giles et al., 2008; Lemmey et al., 2009; Summers et al., 2010; Tournadre et al., 2017). Other forms of SS are also frequently referred to as cachexia (Roubenoff et al., 1992), due to their predominant catabolic component (Masuko, 2014). However, there are marked differences between RS and the other forms of SS (Summers et al., 2010) because of the high inflammatory status which characterizes RS, and the relevant role of the adaptive immune system in this disease.

The prevalence of RS cannot be determined with accuracy, largely due to a lack of consensus in establishing a clear cut-off in body composition for its diagnosis. Besides, the syndrome can pass unnoticed in patients with stable weight resulting from the increase in fat volume, which can mask muscle mass loss (Summers et al., 2010). It has though been estimated to be present in 38% of patients with active rheumatoid arthritis and in 10–20% of those with well-controlled disease (Elkan et al., 2009; Phillips et al., 2009; Summers et al., 2010). The appearance of RS does not carry the same poor prognosis as SS associated to AIDS or cancer. Still, its development impacts both life expectancy and quality of life, particularly due to its association with metabolic syndrome, cardiovascular disease, and weakness, in an independent fashion of disease severity (Kotler, 2000; Walsmith and Roubenoff, 2002; Fukuda et al., 2013).

Why does RS run an independent course from disease activity has been difficult to understand, since the condition is principally unchained by systemic inflammation. It appears that the concurrence of a variety of signals triggers RS, the most relevant of them being inflammation-driven increased metabolism, a reduced anabolic activity, the coexistence of malnutrition, peripheral insulin resistance and lack of exercise. Although RS has not been primarily associated with an impairment of anabolic processes, low IGF-1 has been shown to parallel muscle mass loss in experimental models (Soto et al., 2001; Castillero et al., 2009a) and also in the patients, after adjusting for age, sex, and adiposity (Lemmey et al., 2009; Baker et al., 2015). In addition, it has been proposed that rheumatoid arthritis-associated insulin resistance indirectly promotes muscle wasting, because of the physiological anti-catabolic effect of insulin (Walsmith and Roubenoff, 2002). However, further studies are needed to clarify the involvement of this pathway in RS (Lemmey et al., 2009).

Pro-inflammatory cytokines such as TNF, IL-1β and IL-6, have been proved to exert a critical contribution to the hypercatabolic state found in these patients (Roubenoff, 2014). Although RS pathogenesis is poorly understood, elevated serum levels of these cytokines are known to activate UPS. In experimental cachexia, an upregulation of muscle-specific E3 ubiquitin ligases MuRF-1 and atrogin-1 has been shown, followed by an increased myofibrillar proteolysis, through the activation of NF-κB (Li et al., 2009; Varadhan et al., 2014; Little et al., 2017). These data are consistent with the over-activation of UPS during SS, as has also been found in muscle wasting associated to experimental arthritis (Castillero et al., 2009a; Little et al., 2017). In the same way, the increased activity of FoxO resulting from MSTN-dependent PI3K/Akt inhibition enhances the expression of atrogenes, thus promoting protein catabolism during cachexia (McFarlane et al., 2006); however, the role of this transcriptional regulator in RS is not well defined.

Our group recently conducted studies to look into muscle response to chronic arthritis in a rabbit model which emulates RS. The diseased animals had a reduction in weight and muscle size, and an up-regulation of atrogin-1 both in muscle and in synovium, altogether indicating an increased protein breakdown. Strikingly, there was a paradoxical decrease in MSTN expression, along with a reduction in phospho-STAT-3 levels, which pointed to the existence of a compensatory anabolic activation (Little et al., 2017). This pattern of response suggests that the inflamed muscle could contribute to the process of SS through a mechanism of autocrine atrophy triggered by the release of muscle-derived, pro-inflammatory mediators (Little et al., 2017).

It can be envisaged how devastating the effects of atrogenes can be on the musculature of elderly RS patients (Guadalupe-Grau et al., 2015). Also interesting is that both muscle mass loss and IGF-1 content are remarkably returned to normal in patients with RS following a high intensity training program (Lemmey et al., 2009). This underlines the therapeutic goal of breaking down anabolic resistance in RS. On the other hand, standard antirheumatic therapies should not be expected to prevent RS, since changes in body composition can be observed in patients with low disease activity (Metsios et al., 2007). Previous work in our laboratory disclosed a protective role of celecoxib in the development of sarcopenia in rabbits (Romero et al., 2010). The selective inhibition of COX-2, and COX-2 derived products such as PGE2, yielded a reduction in both systemic inflammation and NF-kB activation, together with an amelioration of weight loss in arthritic rabbits. As suggested by previous studies, the inhibitory effect of NSAIDs on NF-kB signaling might be responsible for the suppression of muscle wasting induced by the activation of the ubiquitin-proteasome pathway (Wyke et al., 2004).

As regards myogenesis impairment, there are no conclusive data about its direct contribution to RS. Low MyoD and myogenin levels have been observed in a rat model of arthritis, arguing for this possibility (de Oliveira Nunes Teixeira et al., 2013). In this regard, both TNF and IFNγ can impair myogenesis, as shown in C2C12 myoblasts and in mouse muscle, where the cytokines suppressed MyoD and myogenin through an NFκB-dependent pathway (Guttridge et al., 2000). By contrast, Castillero and co-workers showed increased expression of these myogenic factors in arthritic rats (Castillero et al., 2009a,b).

### OA sarcopenia

The effect of osteoarthritis (OA) represents a third subset of muscle metabolic adaptive response to systemic inflammation. A variety of factors are involved in the pathogenesis of this complex syndrome, including biomechanical stress, senescence, hormonal status and inflammatory mediators, while the genetic background could also be relevant (Herrero-Beaumont et al., 2009). While all joint tissues are targeted by OA, the initial insult can be localized at any of them, and render different syndromes (Roman-Blas et al., 2016). As the disease progresses into advanced phases, the OA syndrome is usually more uniform, although interspersed flares in relationship with acute injuries, can occur (Castañeda et al., 2014). On the whole, the disease can be considered a protean long-course process, with many possible shifts of phenotype along time.

In the same way, inflammation in OA can take many forms. Typically, there is a low-grade inflammatory status in the OA joint, which can get temporarily higher in response to different triggers, such as capsular sprains, micro-trauma, and the presence of crystals or additional danger associated molecular patterns (DAMPs). All these factors are known to couple innate receptors, such as PI3K and the toll-like family (TLRs) (Gómez et al., 2015). Alternatively, biomechanical sensors can also contribute to these flares, since their signaling network interlinks with major inflammatory pathways.

As regards OA-related sarcopenia, it does not only target the neighboring muscles of affected joints but can also involve the whole SKM. However, the underlying mechanisms regulating joint-muscle crosstalk are not yet fully understood. A close relationship between impaired SKM functions and knee OA has been observed, along with an enhanced expression of FoxO1 reflecting lower muscle strength (Levinger et al., 2016). Since cartilage and SKM cells share some cellular pathways, paracrine communication between them remains conceivable, in addition to the influence of a close anatomical proximity (De Ceuninck et al., 2014). On the other hand, individual factors associated to OA, such as lack of physical activity and obesity can play an indirect role in the pathogenesis of OA-related sarcopenia, while they also account for an increased cardiovascular risk and a shortened life expectancy (Yoshimura et al., 2012). This places adipokines as principal actors in this model of SKM adaptive response to ‘low-grade inflammation’ (Scotece et al., 2011, 2017). Adipokines are hormone-like fat-derived factors which contribute to maintain the low-grade inflammatory status in patients with metabolic syndrome (Gómez et al., 2015). Adiponectin and leptin are by far the best characterized adipokines. They both stimulate glucose uptake and fatty acid oxidation both in muscle and in adipose tissue (Kalinkovich and Livshits, 2017). Of them leptin exerts a pro-inflammatory role and adiponectin is mainly regulatory. While serum levels of adiponectin decrease in relationship with OA, age and obesity, serum leptin is enhanced in patients with OA in parallel with the accumulation of adipose mass (Poonpet and Honsawek, 2014; Kalinkovich and Livshits, 2017). Other relevant adipokines reported to be increased in OA are resistin (Koskinen et al., 2014), which drives pro-inflammatory actions in human SKM (Carey et al., 2006) and chemerin (Ma et al., 2015), which has been shown to inhibit myogenesis and induce adipogenesis in C2C12 myoblasts (Li et al., 2015).

Both OA and sarcopenia are prevalent in the elderly (Felson et al., 1987; Fielding et al., 2011). OA is frequently considered as part of the metabolic syndrome and of senescence (Herrero-Beaumont et al., 2009). The severe peri-articular sarcopenia found in OA could be partly due to inactivity, but also due to low-grade persistent systemic inflammation, which is a feature of both syndromes (Krabbe et al., 2004; Scanzello and Loeser, 2015). In fact, sarcopenic obesity is more closely associated with knee OA than non-sarcopenic obesity, thus supporting the tight relationship between muscle metabolism and inflammation in this disease (Lee et al., 2012).

It has been suggested that sarcopenia could not only be a trigger of OA, but also a worsening factor for its progression (De Ceuninck et al., 2014). Muscle transcription factors associated to inflammation, such as STAT-3 and NFκB, correlate with the grade of joint dysfunction, disability and gait impairment in the patients (Levinger et al., 2011b). Similarly, an inverse correlation has been observed between muscular resistance of hamstrings and serum IL-6 levels in elderly women with OA (Santos et al., 2011), suggesting a role for this cytokine in OA sarcopenia. In fact, Levinger and co-workers found augmented levels of IL-6, STAT-3, SOCS-3 and NFκB, among others, in the vastus lateralis of patients with knee OA (Levinger et al., 2011a). The relevance of IL-6 in OA pathogenesis is further sustained by its association with cartilage loss and radiographic knee OA (Livshits et al., 2009; Stannus et al., 2010). The cytokine could therefore account for a pivotal link between OA and sarcopenia, and provide an attractive therapeutic target, although further research is warranted.

### Sarcopenia associated with idiopathic inflammatory myopathies (IMM)

Finally, a distinct pattern of muscle wasting can be observed in patients with idiopathic inflammatory myopathies (IMM). These systemic autoimmune diseases are characterize by weakness, muscle inflammation (Day et al., 2017) and fat mass gain (Cleary et al., 2015), without an increase in serum CRP levels (Hanisch and Zierz, 2015; Malik et al., 2016). Both the innate and the adaptive immune systems are involved in their pathogenesis. On one hand, there is a deep disturbance in the adaptive immune responses, with activation of auto-reactive cytotoxic T cells along with production of autoantibodies (Dalakas, 2010). On the other, an infiltration of inflammatory cells is responsible for cell death, and disruption of the normal muscle architecture. Local production of cytokines participates in IMM pathogenesis, both promoting cell damage and impairing muscle cell function, although some of them, such as IL-1, TNF or IL-15, appear to have a role in repair stages as well. Therefore, the complexity of muscle cytokine networks deserves especial attention at designing therapeutic strategies (Loell and Lundberg, 2011). Also to underline is the fact that muscle function restriction often persists in the patients in the long-term, despite the achievement of remission with immunosuppressive treatments (Loell et al., 2016). There appears to be therefore a permanent *footprint* of a previous muscle injury, which might also be present in the other three subsets of sarcopenia.

In these heterogeneous muscle disorders myocyte degeneration and necrosis occur mainly due to directed self-reactivity of CD8+ T cells. Sarcolemma disruption provokes the release of myoglobin and creatine kinase (Chargé and Rudnicki, 2004). Antigen-specific cytotoxic T lymphocytes (CTL) migrate through the endothelial wall and directly bind to muscle fibers aberrantly expressing major histocompatibility complex (MHC)-I molecules on their surface, through their T-cell receptors. Upon presentation of muscle antigens, these infiltrating CD8+ T cells undergo clonal expansion (Rayavarapu et al., 2013). Histologically, affected muscles are characterized by perivascular cell infiltration, predominantly consisting of CD8+ T cells invading and surrounding healthy-appearing muscle fibers. Direct cytotoxicity to muscle cells can then take place through the release of perforin granules. Cell infiltration is located in different regions of the muscle fascicles (i.e. interfascicular septae, periphery of the fascicle, epimysium, endomysium, etcetera) depending of the clinical subgroup (Vattemi et al., 2014). Cytokines, such as IFN-γ, IL-1, and TNF released by activated T cells, may enhance MHC class I up-regulation and T-cell cytotoxicity. There is also a shift toward Th17 CD4+ T cell differentiation, which furthers the autoimmune process (Tews and Goebel, 1996). However, as mentioned before, it has been reported that IL-1 and TNF may exert a role in muscle regeneration (Loell and Lundberg, 2011). Some of the above-mentioned cytokines signal through the NF-kB pathway and/or are controlled by this transcription factor, which indeed seems to have a key pathogenic role in IIM. It is well known that NF-kB becomes activated both in inflammatory cells and in myocytes and enhances MHC-I expression in muscle fibers. This event has been associated with ER stress, which fuels MSTN production and muscle injury (Tews and Goebel, 1996; Nagaraju et al., 2005; Nogalska et al., 2007; Creus et al., 2009). As has been previously mentioned, NF-kB is known to induce atrogene expression and loss of MyoD messenger RNA (Guttridge et al., 2000; Cai et al., 2004). On the other hand, MSTN exerts important anti-anabolic effects by blocking the PI3k/Akt/mTOR signaling pathway (Morissette et al., 2009; Schiaffino et al., 2013; Brooks and Myburgh, 2014; Retamales et al., 2015). In conclusion, these pro-catabolic and anti-anabolic mediators can elicit IIM-dependent sarcopenia.

## The dual role of JAK/STAT and IL-6 in SKM

While the JAK/STAT pathway has been reported to play a crucial role in myogenesis, its precise contribution to muscle wasting and regeneration is yet to be defined. According to results drawn by different studies, it can be argued that the activation of JAK/STAT results in different effects in healthy and in injured muscle cells. This is likely due to the magnitude and duration of the induction.

As previously mentioned, the IL-6/STAT-3/SOCS-3 axis contributes to myogenic differentiation in physiological situations (Hoene et al., 2013). By contrast, experimental murine models of rheumatoid cachexia exhibit a profound total and muscle and total weight loss and elevated IL-6 levels are consistently found along time. IL-6 involvement could be determinant to muscle atrophy (Bonetto et al., 2012), and drive muscle wasting through the activation of UPS (Bodell et al., 2009). Furthermore, chronically elevated IL-6 levels might reflect a feedback mechanism triggered by an impairment in IL-6 dependent signaling. To our knowledge, whether this phenomenon of ‘IL-6 resistance’ takes place during muscle wasting has not been addressed. However, it is conceivable that IL-6 deregulation during disease could follow a similar pattern to those of insulin or leptin tissue resistance.

Hypothetically, during RS this phenomenon could be due to an overexpression of SOCS-3 in muscle cells. Consistently, muscle SOCS-3 overexpression is known to abrogate leptin-dependent STAT-3 phosphorylation, in this way favoring leptin resistance (Bjørbaek et al., 1998). Interestingly, increases in SOCS-3 could also inhibit the insulin receptor, thus promoting insulin resistance as well (Ueki et al., 2004). Also of interest would be the assessment of soluble gp130/sIL-6R in RS muscle, since this decoy receptor could account for an additional mechanism of IL-6 hyporesponsiveness (Jostock et al., 2001). The conflicting effects of IL-6 on muscle certainly represent a novel avenue for further research, which could be termed the ‘*IL-6 paradox*’.

In line with this findings, Tierney et al. and Price et al. reported that the enhancement of JAK2/STAT-3 signaling impaired myogenesis in aging mice, likely due to a loss of SC self-renewal capability (Tierney et al., 2014; Price et al., 2015). Subsequently, STAT-3 has been hold responsible for driving SS. Bonetto and co-workers found that STAT-3 and its responsive genes were up-regulated in mice with cancer-associated cachexia (Bonetto et al., 2011). In these mice, they found that constitutive activation of the transcription factor worsened their wasting status, and that JAK1/2 or STAT-3 blockade could revert this situation (Bonetto et al., 2012). As has been shown in transfected C2C12 myoblasts, overexpression of STAT-3 results in its direct interact with MyoD, thus inhibiting myogenic differentiation. Reciprocally, MyoD was shown to decrease STAT-3 activity (Kataoka et al., 2003).

Although there are scant studies looking into this signaling cascade in OA related sarcopenia, a role of the IL-6/STAT-3/SOCS-3 axis in muscle wasting following ‘chronic low-grade inflammation’ can be foreseen. Of note, in addition to experimental findings, levels of these mediators were increased in the vastus lateralis of patients with knee OA (Levinger et al., 2011a).

Globally, these data point to a role of STAT-3 in normal regeneration of healthy muscle. Nonetheless, its chronic activation during aging or chronic inflammation could carry an impairment of SC self-renewal, a lower proliferation of myoblasts and an overall abnormal muscle repair.

Among all the JAK and STAT molecular mediators, STAT-3 has received major attention in the study of healthy and damaged muscle. This transcription factor appears, nonetheless, to play distinct roles on myogenesis depending on the JAK protein kinase activated upstream. In brief, the JAK2/STAT2/STAT-3 pathway has been found to exert a pro-differentiation effect in primary muscle cells and C2C12 myoblasts (Wang et al., 2008), whereas JAK1/STAT-1/STAT-3 was shown to boost myoblast proliferation through the regulation of cell cycle-associated genes' expression. As reported, the latter could also prevent myoblast premature differentiation, thus acting as a checkpoint during myogenesis (Sun et al., 2007). In a subsequent study, the same group observed that SOCS-1 and SOCS-3 intercepted JAK1/STAT-1/STAT-3 signaling, thereby promoting myogenic differentiation (Diao et al., 2009).

Very little is known about JAK/STAT activation in IIM. According to Illa and co-workers patients with dermatomyositis could show an increased STAT-1 activation in myofibers (Illa et al., 1997). Presumably, the enhancement of JAK/STAT dependent transcription by local cytokine networks drives muscle wasting in IIM. The overall extent of muscle wasting is likely determined by activation of specific JAKs and STATs triggered by a particular group of cytokines. Whether these changes can be reverted with treatment, as well as the effects of different JAK inhibitors depending on their specificity for particular JAK/STAT cascades, deserves further research.

## Conclusions and future perspectives

A tight regulation of protein turnover and cell growth is crucial to maintain homeostasis in SKM. Chronic systemic inflammation, however, provokes a dramatical shift in this balance, thus compromising SKM mass. Behind this fine-tuning, there is a very intricate network of catabolic and anabolic signaling pathways, which are summarized in Figure 1. In short, induction of the Akt/mTOR pathway not only enhances myogenesis and protein synthesis, but also slows down catabolic UPS and the autophagic machinery. Conversely, MSTN exerts important anti-anabolic and pro-catabolic effects, through the suppression of the Akt/mTOR axis and the induction of UPS, respectively. Pro-inflammatory cytokines mostly act as pro-catabolic and anti-anabolic factors, with IL-6 exerting a dual role in SKM turnover.

It has not been until recently that the pathogenesis of chronic inflammation-related sarcopenia has been understood. Consequently, the SKM response to the inflammatory milieu is often unadvertised in the assessment of prevalent conditions. In addition, despite the recent advances providing insight on the relationship between chronic inflammation and sarcopenia, there are still substantial gaps, which may account for the lack of treatments for sarcopenia. Intriguingly, muscle mass loss does not seem to revert despite the achievement of remission. Therefore, the mechanisms behind this permanent *footprint* need to be approached in future studies.

While innate and adaptive immune responses are extremely energy-consuming, metabolic inflammation does not lead to an increased energy expenditure. Notwithstanding this fact, metabolic inflammation is often said to resemble a smoldering fire, which is difficult to extinguish (Hotamisligil, 2006). Moreover, chronic low-grade inflammation and metabolic dysfunction drive the development of the most prevalent chronic diseases, particularly those targeting the musculoskeletal system such as OA (Hotamisligil, 2006; Robinson et al., 2016). Of interest, they also significantly contribute to the fragility syndrome of the elderly. As is widely acknowledged, major factors associated with accelerated atherosclerosis are the components of the metabolic cluster, namely hypertension, high blood glucose, excess in waist fat, and abnormally increased lipid levels. Obesity, which shows a higher prevalence in OA than in non-OA age-matched individuals, could be the most relevant of these traits.

Additional pathways of muscle loss are unchained by anorexia, asthenia and inactivity, all of which are typical features of chronic systemic inflammation (Phillips et al., 2009). These processes are, in fact, shared by the four previously mentioned clinical settings (Huffman et al., 2017). In this regard, as has been recently reviewed in depth by Dalle and coworkers, exercise and dietary interventions have proved beneficial against the anabolic resistance of the elderly (Dalle et al., 2017). On the other hand, considering the impact of chronic inflammation on the development of this resistance, it appears that anti-inflammatory therapies could provide an extra benefit to lifestyle and nutrition changes on restoring muscle mass in the aged population (Dalle et al., 2017). Similarly, not only suppression of disease activity but also exercise and a well-suited nutritional management have been underlined as major strategies against RS (Masuko, 2014).

An increasing attention is being currently paid to the JAK/STAT pathway as a promising target for the treatment of muscle wasting diseases. The product of this signaling cascade seems to differ, likely depending on the magnitude and duration of the input. It would be of high interest to assess therapeutic effects of JAK/STAT inhibitors not only on inflammatory parameters, but also on muscle mass and function both in clinical and preclinical studies, since they certainly stand as promising drugs in the management of sarcopenia.

On the whole, to look into muscle involvement in distinct types of systemic conditions may help identifying patterns of muscle classic inflammatory response (i.e. IMM), low-grade chronic inflammation (i.e. PS or OA) or severe chronic systemic inflammation (i.e. RS).

## Author contributions

SP-B, RL and GH-B were in charge with conception and design; SP-B, IP-P, and GH-B: were involved in drafting the article; SP-B, IP-P, JR-B, OS-P, RL, and GH-B: revise it critically for important intellectual content and approved the final version to be published.

### Conflict of interest statement

The authors declare that the research was conducted in the absence of any commercial or financial relationships that could be construed as a potential conflict of interest.

## Abbreviations

PDK-1: 3′-Phoshphoinositide-Dependent Protein Kinase-1
4E-BP1: 4E-Binding Protein 1
PRR: Activate Pattern Recognition Receptors
ActRII: Activin Type II Receptors
ADM: Amyopathic Dermatomyositis
ATG: Autophagy-Related Genes
CRP: C-Reactive Protein
CA: Chronic Arthritis
ER: Endoplasmic Reticulum
ERK: Extracellular Signal–Regulated Kinase
FoxO: Forkhead O
GDF-8: Growth Differentiation Factor-8
GH: Growth Hormone
IMM: Idiopathic Inflammatory Myopathies
IGF: Insulin Growth Factor
IRS-1: Insulin Receptor Substrate-1
IFN: Interferon
IL: Interleukin
JAK: Janus Kinase
MHC: Major Histocompatibility Complex
mTOR: Mammalian Target of Rapamycin
MAPK: Mitogen Activated Protein Kinases
MAFbx: Muscle Atrophy F-Box Protein
MuRF-1: Muscle Ring Finger-1
MyoD: Myogenic Differentiation
Myf5: Myogenic Factor 5
MSTN: Myostatin
NFκB: Nuclear Factor KB
OA: Osteoarthritis
70S6K: P70-S6 Kinase
Pax7: Paired Box Transcription Factor 7
PI3K: Phosphatidyilinositol 3 Kinase
PS: Primary Sarcopenia
RA: Rheumatoid Arthritis
RS: Rheumatoid Sarcopenia
RS6K: Ribosomal Protein S6 Kinase Beta-1
SC: Satellite Cells
SS: Secondary Sarcopenia
STAT: Signal Transducer and Activation Of Transcription
SKM: Skeletal Muscle
TLR: Toll-Like Receptors
TGF: Transforming Growth Factor
TNF: Tumor Necrosis Factor
UPS: Ubiquitin-Proteasome System
